# Nanocolumnar Crystalline Vanadium Oxide-Molybdenum Oxide Antireflective Smart Thin Films with Superior Nanomechanical Properties

**DOI:** 10.1038/srep36811

**Published:** 2016-11-17

**Authors:** Arjun Dey, Manish Kumar Nayak, A. Carmel Mary Esther, Maurya Sandeep Pradeepkumar, Deeksha Porwal, A. K. Gupta, Parthasarathi Bera, Harish C. Barshilia, Anoop Kumar Mukhopadhyay, Ajoy Kumar Pandey, Kallol Khan, Manjima Bhattacharya, D. Raghavendra Kumar, N. Sridhara, Anand Kumar Sharma

**Affiliations:** 1 ISRO Satellite Centre, Bangalore 560017, India; 2 Department of Metallurgical and Materials Engineering, National Institute of Technology, Warangal-506004, Telengana, India; 3 Department of Mechanical Engineering, National Institute of Technology, Durgapur, West Bengal 713 209, India; 4 Surface Engineering Division, CSIR-National Aerospace Laboratories, Bangalore 560 017, India; 5 Advanced Mechanical and Materials Characterization Division, CSIR-Central Glass and Ceramic Research Institute, Kolkata 700032, India

## Abstract

Vanadium oxide-molybdenum oxide (VO-MO) thin (21–475 nm) films were grown on quartz and silicon substrates by pulsed RF magnetron sputtering technique by altering the RF power from 100 to 600 W. Crystalline VO-MO thin films showed the mixed phases of vanadium oxides e.g., V_2_O_5_, V_2_O_3_ and VO_2_ along with MoO_3_. Reversible or smart transition was found to occur just above the room temperature i.e., at ~45–50 °C. The VO-MO films deposited on quartz showed a gradual decrease in transmittance with increase in film thickness. But, the VO-MO films on silicon exhibited reflectance that was significantly lower than that of the substrate. Further, the effect of low temperature (i.e., 100 °C) vacuum (10^−5^ mbar) annealing on optical properties e.g., solar absorptance, transmittance and reflectance as well as the optical constants e.g., optical band gap, refractive index and extinction coefficient were studied. Sheet resistance, oxidation state and nanomechanical properties e.g., nanohardness and elastic modulus of the VO-MO thin films were also investigated in as-deposited condition as well as after the vacuum annealing treatment. Finally, the combination of the nanoindentation technique and the finite element modeling (FEM) was employed to investigate yield stress and von Mises stress distribution of the VO-MO thin films.

Vanadium oxides based films and coatings are extensively studied due to both thermochromic^1^,^2^,^3^,^4^ and electrochromic^5^,^6^ characteristic, catalytic behaviours^7^,^8^ etc. Different oxide states of vanadium viz. V_2_O_5_^9^,^10^,^11^, V_2_O_3_^12^,^13^, VO_2_^1^,^2^,^6^, VO^12^,^13^ etc. show reversible phase transition characteristics with a drastic alteration in the optical, electrical and thermal behaviours. Among all the aforesaid oxides, VO_2_ and V_2_O_5_ are extensively investigated owing to the passion of positive phase transition temperatures. The tuning of transition temperature of vanadium oxide is usually achieved by doping/adding second phase with other transition metals e.g., both higher and lower valent metals such as Mo^14^,^15^,^16^,^17^,^18^,^19^,^20^,^21^,^22^,^23^,^24^,^25^,^26^, W^27^, Mn^15^, Ti^28^,^29^, Nb^26^,^30^, Cr^30^ and noble metal i.e., Au^31^ as well. After doping/adding second phase, the transition temperature of vanadium oxide is reported to be decreased^14^,^15^,^17^,^18^,^19^,^20^,^24^,^25^,^31^.

The Mo and/or molybdenum oxide doped vanadium oxides are reported to be grown by a multitude of techniques such as magnetron sputtering technique^15^, atmospheric pressure chemical vapour deposition^26^, cathodic elctrodeposition^16^, sol-gel^14^,^17^,^20^,^21^,^24^, hydrothermal synthesis^19^,^21^, combustion synthesis technique^18^, spray pyrolysis^25^ and electron beam evaporation techniques^22^,^23^. In general, the introduction of Mo or oxides of Mo are reported to have assisted in various extents of reduction in the transition temperature of VO_2_ e.g., from about 68 °C^32^ to 55–30 °C^17^,^24^, 50 °C^25^, 53–(−)91 °C^15^, 47.5–24 °C^14^ and 25 °C^19^. The optical properties such as transmittance^15^,^16^,^21^,^22^,^23^,^24^,^25^, reflectance^14^ and electrical conductivity of these coatings are also extensively studied^14^,^15^,^18^,^20^,^21^,^22^,^23^. For instance, the electrochromic behaviour is investigated by Jin *et al.*^16^. Additional efforts are also directed towards the dissociation^14^,^15^,^17^,^18^,^20^ induced reduction of the V_2_O_5_ phase as well as enhancement in crystallinity^15^ through vacuum annealing at high temperature e.g., 450–500 °C. There is only a lone attempt^33^ to reduce the vacuum annealing temperature of pure i.e., un-doped vanadium oxide system further downwards to e.g., 200–500 °C. Thus, the low temperature annealing of VO-MO system is yet to be extensively explored. As a consequence, the systematic investigations of microstructural, electronic, optical, electrical and mechanical behaviours of VO-MO thin films in as deposited condition as well as following the low temperature e.g., 100 °C vacuum annealing treatment are not yet attempted.

Thus, in the present study, VO-MO thin films are grown on both quartz and silicon substrates by pulsed radio frequency (RF) magnetron sputtering technique at different thicknesses in the range of ~21–475 nm by altering RF power from 100 to 600 W. Microstructural characterization of the VO-MO thin films on quartz are carried out by field emission scanning electron microscopy (FESEM), atomic force microscopy (AFM) and transmission electron microscopy (TEM). Surface characteristic of the deposited thin film is investigated by water contact angle (WCA) measurement technique. X-ray diffraction (XRD) and X-ray photoelectron spectroscopy (XPS) are employed to analyse the phase and electronic structures of the thin films. Thermo-optical properties viz. solar transmittance (τ_s_), reflectance (ρ_s_), absorptance (α_s_) and IR emittance (ε_IR_) along with optical constants e.g., optical band gap, refractive index and extinction coefficient of VO-MO thin films are evaluated. Sheet resistance (R_s_) of the thin films is also measured by the two probe method. Phase transition behaviour of the VO-MO thin film is evaluated by differential scanning calorimetry (DSC) and temperature dependent resistance measurement techniques. Particularly, the mechanical properties of the VO-MO thin film at microstructural length scale are also investigated. The combination of the nanoindentation technique and the FEM is utilized to evaluate nanohardness, Young’s modulus, yield stress and von Mises stress distribution of the VO-MO thin films. Further, the effect of low temperature vacuum annealing (100 °C, 10^−5^ mbar) on microstructural, thermo-optical, electrical, electronic, and nanomechanical behaviour of VO-MO thin films are systematically studied. The VO-MO thin films grown on silicon substrate are also investigated for their antireflection property.

## Materials and Methods

In the present study, VO-MO thin films were grown on quartz and silicon substrates at room temperature by pulsed RF magnetron sputtering (SD20, Scientific vacuum systems, UK) technique. The quartz substrate (40 × 40 × 0.2 mm^3^) was obtained from Astro Optics, India and silicon wafer (~40 × 35 × 2 mm^3^) was procured from Silicon Valley Microelectronics Inc., USA. A high purity (99.995%) V_2_O_5_ target (Vin Karola Instruments, USA) of 8 inch diameter and 3 mm thickness was used for the development of the VO-MO thin films. The target was bonded with a Cu backup. A thin molybdenum strip (Vin Karola Instruments, USA) of 180 × 18 × 1 mm^3^ size was placed along with the V_2_O_5_ target to co-sputter the VO-MO film. Ultra-pure argon gas (~99.9998%, Praxair, India) was utilized to produce plasma for the deposition of the thin films. The distance between the target and the substrate was kept constant at 140 mm. The pulse frequency was set at 100 Hz with a fixed duty cycle of 57%. Prior to the deposition process, utilizing a combination of both rotary and turbo molecular pumps the vacuum chamber was evacuated to a pressure of better than 5 × 10^−6^ mbar. Though, the working pressure for deposition was set to1.5 × 10^−2^ mbar, prior to deposition of films, pre-sputtering was performed for 10 min to reduce the contamination, if any. The RF power during the film deposition was altered from 100 to 600 W. Six different RF powers were chosen with a constant increment of 100 W. The duration of the deposition time of the film was kept constant at 1 hour.

To examine if after vacuum annealing there was any alteration of the oxidation state, thermo-optical, electrical and nanomechanical properties, the deposited VO-MO films were subsequently annealed. The annealing was done in vacuum (10^−5^ mbar) at a low temperature of 100 °C for 1 hour. A custom made high vacuum horizontal furnace (Hind High Vacuum Pvt. Ltd., Bengaluru, India) was used for this purpose.

Both FESEM (Supra VP 40, Carl Zeiss, Germany) and cross sectional TEM (Tecnai G^2^ 30, S-Twin, 300 kV, FEI, The Netherlands) techniques were used to investigate the microstructure of the VO-MO thin films. The energy dispersive X-ray (EDX) spectra of the VO-MO thin films were acquired utilizing the corresponding customised units attached to the machines for the FESEM and TEM studies. The surface morphologies of the VO-MO thin films were investigated by using the AFM (CSEM, USA) technique.

The thicknesses of the VO-MO thin films were measured by using a surface profilometer (Nanomap 500 LS 3D, USA). To study the nature of surface of the thin films, the sessile drop (vol.: 10 μl, dispensing rate: 15 μl.min^−1^) method (ACamD2, Apex Instruments Co. Pvt. Ltd., Kolkata, India) was utilized to measure WCA using the conventional half-angle fitting method. For the VO-MO films deposited on quartz substrates the tests were performed with distilled water at room temperature and at atmospheric pressure.

Phase analysis of the VO-MO thin films was carried out by employing XRD (PANalytical X’pert Pro MPD diffractometer, The Netherlands) technique using monochromatic Cu Kα1 radiation (λ = 0.154058 nm), 35 mA, 40 kV with a very small step size of 0.03°. The crystallite size of the VO-MO thin films was calculated from the diffraction peaks by using the Debye-Seherrer relation.

XPS of the VO−MO thin films grown at both low (e.g., 130 nm at 200 W) and high (e.g., 475 nm at 600 W) RF power and subsequently annealed at a specifically low temperature of 100 °C were recorded with a SPECS spectrometer using an X-ray source of non-monochromatic AlKα radiation (1486.6 eV) operated at 150 W (12 kV, 12.5 mA). The binding energies reported here were referenced with O1s peak at 530.0 eV^9^. All the survey spectra were obtained with pass energy of 70 eV with a step increment of 0.5 eV, whereas individual spectra were recorded with pass energy and step increment of 25 and 0.05 eV, respectively. V2p and O1s components were curve-fitted with Gaussian-Lorentzian peaks after Shirley background subtraction employing CasaXPS program. As per ASTM C1371–04a standard the average ε_IR_ values of the VO-MO thin films were measured in the wavelength range of 3–30 μm by utilizing an emissometer (AE, Devices and Services Co., USA).

DSC (Q100, TA Instruments, USA) technique in helium environment was utilized to investigate phase transition behaviour of the VO-MO thin films. The heating and cooling rate was kept fixed at 10 °C.min^−1^. At least 3 heating-cooling cycles were performed to check out the reproducibility of the reversible phase transition behaviour of the VO-MO thin films.

Spectra of τ_s_ and ρ_s_ of the VO-MO thin films as a function of wavelength were recorded utilizing UV-VIS-NIR spectrophotometer (Cary 5000, Agilent Technologies, USA) in solar region (200 nm to 2300 nm) of the spectral window. As per ASTM C1549-09 standard the average values of α_s,_ τ_s_ and ρ_s_ of the VO-MO thin films were measured in the wavelength range of 200–2500 nm under the ambient condition by utilizing a solar spectrum reflectometer (SSR-E, Devices and Services Co., USA).

The optical absorption coefficient (α) of the VO-MO thin films was calculated from the experimentally measured film thickness and optical transmittance data^34^. The optical band gap of the of the VO-MO thin films was calculated by the conventional ‘Tauc extrapolation’ method from the transmittance spectra^34^,^35^. Here the best fitting was found for only the direct allowed transition. Further, the refractive index (n) of the of the VO-MO thin films was calculated by the ‘envelope method’ from the subsequent maxima of reflectance spectra^34^,^35^. The n values were taken as 1.45 and 3.42 for quartz and silicon substrates, respectively. Thickness (t) of the VO-MO thin films was also theoretically predicted by applying the following equation (1)^34^,^35^:


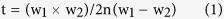


In equation (1) w_1_ and w_2_ are the wavelengths corresponding to two successive maxima of the reflectance spectra of the VO-MO thin films. Finally, the extinction coefficient (k) of the films was determined using the absorption coefficient data^34^,^35^.

As per ASTM D 257-9, the R_s_ data of the VO-MO thin films deposited on quartz substrates was measured by the two-probe resistance meter (Trek Model 152-1, Trek Inc., USA). Further, the temperature dependent R_s_ measurement was carried out by resistive meter with cylindrical four point probe head along with multi-height facility (Model RM 3000, Jandal Engineering Ltd., UK).

The nanoindentation (Fischerscope H100-XYp, Fischer, Switzerland) experiments were conducted at 1.5 mN with Berkovich diamond indenter on both as-deposited and annealed VO-MO thin films deposited on quartz substrates. The thicker film is grown judiciously to avoid the substrate effect, if any. Both loading and unloading times were kept constant at 30 s. The Oliver-Pharr method^36^ was used to evaluate the nanohardness (H) and Young’s modulus (E) of the VO-MO thin films. To report the average data of H and E of the VO-MO thin films, at least 16 (4 × 4 array) indents were performed on locations chosen randomly without any particular bias. Further, using the combinational approach the plastic properties like yield stress (*σ*_*y*_) and strain hardening exponent (*n*) of as-deposited and annealed VO-MO thin films were investigated. It involved the experimentally obtained load-depth (i.e., P–h) curve and the FEM based simulation of the same. The iterative modification of the computed curve was continued, until the particular combination of elastic-plastic properties that lead to a very close agreement between the experimentally obtained and the simulated P-h plots was attained. The details of FE model of the nanoindentation are discussed elsewhere^37^.

## Results and Discussion

### As-deposited VO-MO films

#### Microstructural study

**Figure 1:**
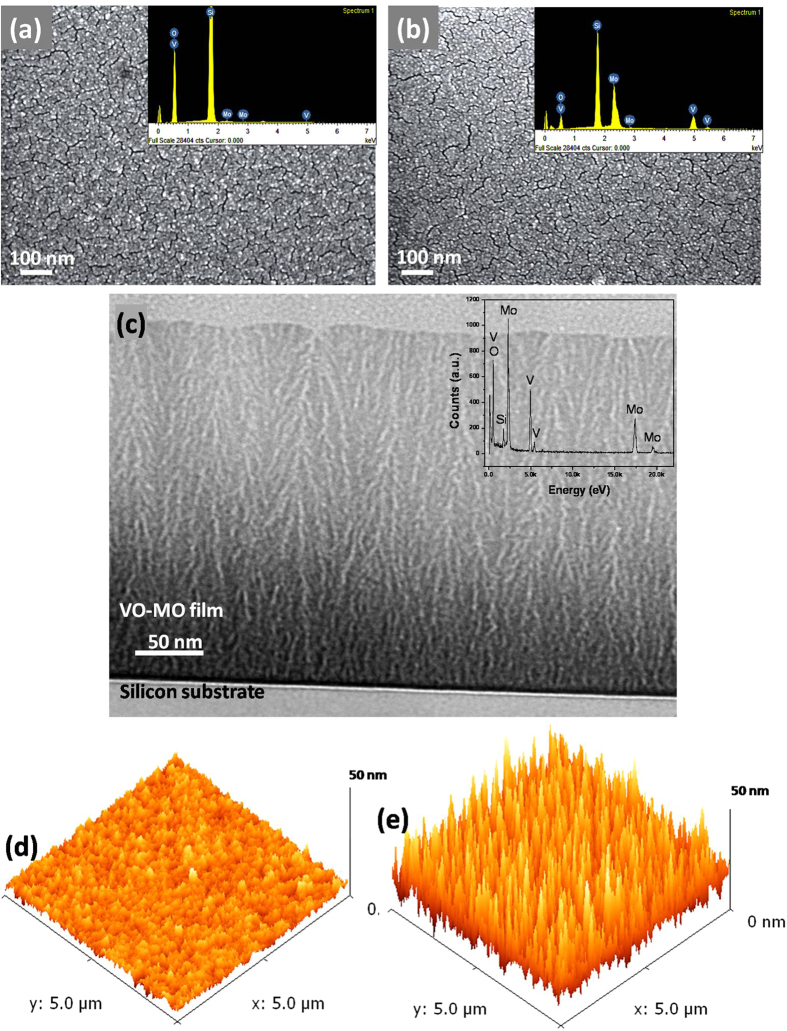
FESEM photomicrographs of VO-MO thin films grown on quartz: typical lower thickness e.g., (**a**) 100 W/21 nm and higher thickness e.g., (**b**) 400 W/382 nm (Insets: corresponding EDX spectra.). (**c**) The typical bright field cross sectional TEM image of VO-MO film on silicon substrate with corresponding EDX (inset). AFM images of VO-MO thin films grown on quartz: typical lower thickness e.g., (**d**) 200 W/130 nm and higher thickness e.g., (**e**) 600 W/475 nm.

The FESEM photomicrograph of the VO-MO thin film deposited on quartz substrates at a low RF power of 100 W is shown in Fig. 1(a). A similar FESEM photomicrograph for the VO-MO thin films deposited on quartz substrates at a high RF power of 400 W is shown in Fig. 1(b). Both the VO-MO thin films showed uniform, smooth and grainy surface morphology. The corresponding EDX spectra are appended as insets of Fig. 1(a,b) in turn. As expected the EDX data (Fig. 1(a,b), insets) confirmed the presence of vanadium, molybdenum and oxygen as the main constituents. The additional sharp peak of silicon (Fig. 1(a,b), insets) was observed due to the quartz substrate.

The bright field cross sectional TEM image of the VO-MO thin film deposited on silicon substrate is presented in Fig. 1(c). The corresponding EDX data is shown as inset of Fig. 1(c). The nanocolumnar structure of the film is established from the TEM photomicrograph, Fig. 1(c). The film substrate interface showed good adhesion without any evidence of delamination. The high precision EDX data shown as inset of Fig. 1(c) confirmed the presence of only vanadium, molybdenum, oxygen and silicon (i.e., from substrate). These data corroborated well also with the FESEM observations (Fig. 1(a,b), insets).

The AFM images of the relatively thin and relatively thick VO-MO films deposited on quartz substrates at RF powers of 200 and 600 W are shown respectively, in Fig. 1(d,e). Both the films showed uniform morphology but the thicker film (Fig. 1e) had surface roughness (R_a_) higher then that of the thinner (Fig. 1d) film.

**Figure 2:**
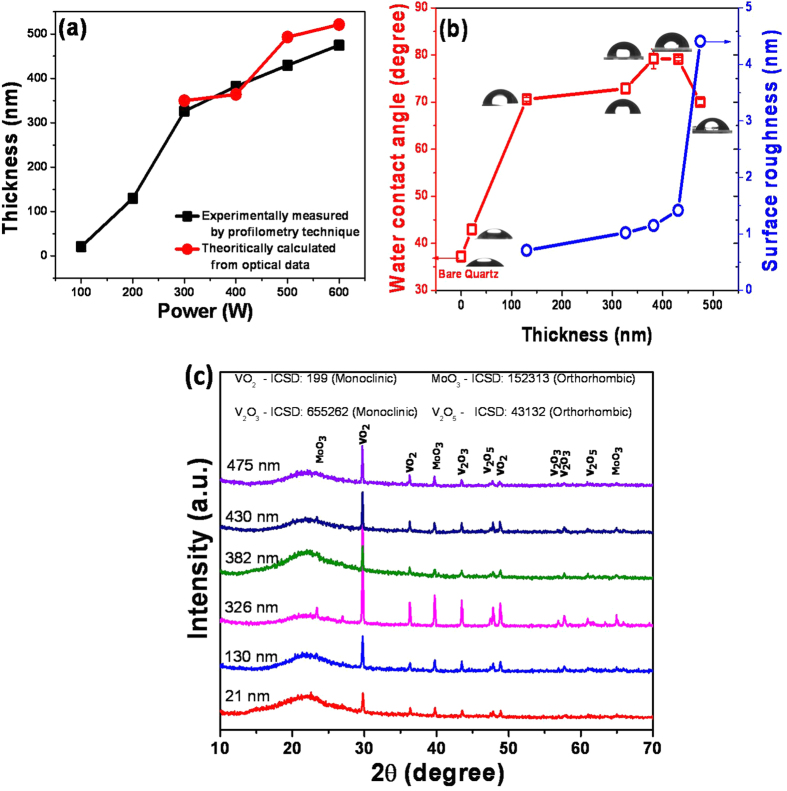
Variation of (**a**) thickness of VO-MO films as the function of RF power (**b**) surface roughness vs. water contact angle as the function of thickness and (**c**) XRD patterns of VO-MO films.

Based on nanoprofilometry, the change in thickness of the VO-MO thin films is shown in Fig. 2(a) as a function of RF power. The increase in thickness e.g., from ~21 nm to ~475 nm is observed with increase in RF power from 100 W to 600 W. The increase in RF power leads to boost in the deposition rate. Thus, the constant deposition duration (i.e., 1 hour) results an increase in film thickness^9^.

Further, the variation of R_a_ as a function of film thickness is shown in Fig. 2(b). It is observed that the R_a_ of VO-MO thin films was increased marginally from 0.71 nm to 1.42 nm with increase in thickness from ~130 nm to ~430 nm. However, beyond ~430 nm the increase was significant e.g., R_a_ ~4.41 at the thickness of ~475 nm.

The experimentally measured data on variation of WCA as a function of the VO-MO film thickness are also shown in Fig. 2(b). The WCA value of bare quartz substrate was measured as ~37° which had marginally increased to e.g., ~43° for the thinnest (21 nm) VO-MO film. However, beyond this thickness the WCA was significantly increased up to as high as e.g., 79° (Fig. 2b). The lower value of WCA measured for the thinnest VO-MO film (Fig. 2b) could be due to the dominant influence of the relatively smoother substrate. It seems plausible to argue that the increase in WCA with increase in R_a_ (Fig. 2b) was possibly linked to the increase in the heights of several adjacent asperities which had covered the corresponding area on the respective film surfaces.

#### Phase analysis

XRD patterns of VO-MO thin films are shown in Fig. 2(c). The data presented in Fig. 2(c) confirmed the presence of crystalline phases of mixed vanadium oxide of different oxidation state^9^,^15^,^20^,^24^,^38^ e.g., V_2_O_5_, V_2_O_3_ and VO_2_ and molybdenum oxide. The patterns exhibited eleven peaks in the 2θ range of 10–70° corresponding to the VO_2_: ICSD-199, V_2_O_5_: ICSD-43132, V_2_O_3_: ICSD-655262 and MoO_3_: ICSD-152313. As the thickness increased from 21 nm to 475 nm, the intensity of the peaks increased but position of the peak remained at the same diffraction angles (Fig. 2c). This fact confirmed further that the same phases were present in the VO-MO films of various thicknesses.

#### Phase transition behaviour by DSC and temperature dependent R_s_ measurement

**Figure 3:**
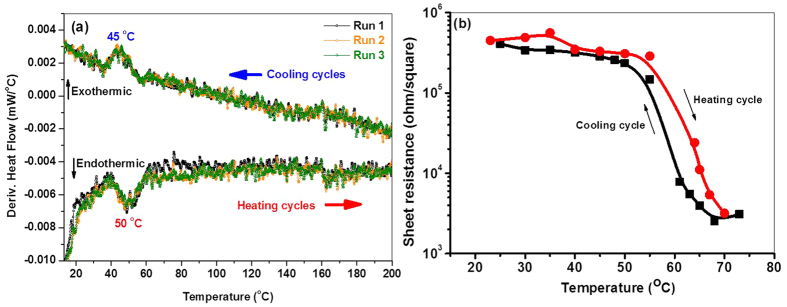
(**a**) DSC curves and (**b**) temperature dependent R_s_ of VO-MO film grown on quartz at 500 W/430 nm.

The DSC curves and the temperature dependent R_s_ data plots of the VO-MO thin films are shown in Fig. 3(a,b), respectively. The VO-MO thin films showed prominent signatures of smart phase transitions which had appeared during both heating and cooling cycles. The transition showed a minor hysteresis, Fig. 3(a). In other words, during the heating cycle an endothermic peak had occurred at ~50 °C but during the cooling cycle an exothermic peak had occurred at ~45 °C. The reproducibility of the smart phase transitions in the VO-MO thin films was confirmed by the corresponding DSC data (Fig. 3a) obtained from at least three consecutive runs of heating-cooling cycles.

For the VO-MO thin film the experimentally measured data on temperature dependence of R_s_ are shown in Fig. 3(b). A drastic alteration in R_s_ value (i.e., from mega to kilo ohm and vice versa) had definitely occurred beyond the transition temperature of ~50 °C. Similar to the case of the DSC data, Fig. 3(a), the drastic alteration in R_s_ also showed a minor hysteresis, Fig. 3(b). Thus, the DSC and temperature dependent electrical resistance data, Fig. 3(a,b), confirmed the reversible or smart phase transition characteristics of the present VO-MO thin film.

#### Transmittance and reflectance properties

**Figure 4:**
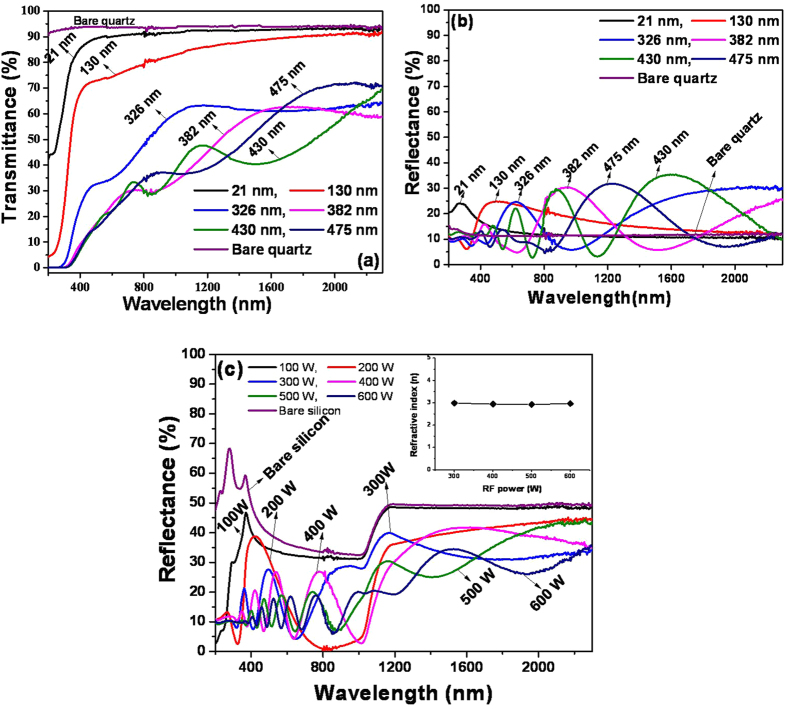
(**a**) Transmittance (**b**) reflectance spectra of VO-MO films on quartz and (**c**) reflectance spectra of VO-MO films on silicon as a function of wavelength (inset: variation of refractive index of VO-MO films on silicon as a function of RF power).

The transmittance spectra of the VO-MO thin films on quartz substrates are shown as a function of wavelength from 200 nm to 2300 nm in Fig. 4(a). The corresponding reflectance spectra are shown in Fig. 4(b). Similar reflectance spectra of the co-deposited VO-MO thin films on silicon substrates are shown in Fig. 4(c) as a function of the same range of wavelength as shown in Fig. 4(a,b). The reflectance data of bare quartz and silicon substrates are in turn included in Fig. 4(b,c), for the purpose of comparison only.

A significant reduction in transmittance occurred with increase in the film thickness from ~21 to ~475 nm, Fig. 4(a). Porwal *et al.*^9^ and Mlyuka *et al.*^29^ have also reported the decrease in transmittance with increase in thickness of the VO films. The fundamental absorption edge was shifted to the higher wavelength e.g., from ~360 nm to ~470 nm. In the present work, this shift was linked to the increase in R_a_ with increase in the film thickness. It is therefore plausible to argue that the increase in crystallinity caused higher surface roughness^39^,^40^,^41^ as well as grain growth^42^,^43^ and hence, contributed to enhance the scattering loss. The enhanced scattering loss, *in turn*, had ultimately resulted in lower transmittance, Fig. 4(a), of the VO-MO thin films. On the other hand, especially for the relatively thicker (e.g., 326–475 nm) VO-MO films several maxima and minima had occurred in the corresponding reflectance spectra, Fig. 4(b).

#### Antireflective properties

As expected the bare silicon substrate showed opaque behaviour in UV-VIS region while it was transparent (~53–54%) in NIR. This unique behaviour makes silicon a potential candidate for versatile applications in opto-electronic, energy harvesting, IR detector etc. fields^44^,^45^. It is very interesting to note in this context that the reflectance data of the VO-MO coated silicon substrates were always lower than that the bare silicon substrate, Fig. 4(c). Thus, the data presented in Fig. 4(c) confirmed the strong antireflection characteristics of the VO-MO thin films. Further, the antireflection behaviour can assist to enhance the efficiency and functionality properties of the aforesaid applications.

Moreover, the reflectance spectra of the 300 to 600 W RF power deposited VO-MO thin films exhibited several maxima and minima, Fig. 4(c). It was evident from the data presented in Fig. 4(c) that the VO-MO thin films grown at different RF powers of viz.100–600 W on silicon substrates had exhibited the lowest reflectance values of e.g., 2.7% at 200 nm, 6.84% at 640 nm, 4.18% at 650 nm, 0.98% at 795 nm, 5.97% at 860 nm and 2.8% at 1010 nm. It has been reported that the V_2_O_5_/silicon system shows the reflectance value of ~20% in wavelength of 500–1100 nm region^44^. Although the experimental conditions are not exactly similar, the experimental data of the present work (Fig. 4c) confirmed that the VO-MO thin films deposited on silicon substrates had exhibited reflectance value much lower than that reported for the V_2_O_5_/silicon system^44^.

It seems therefore plausible to suggest that due to the superior antireflection property (Fig. 4c), the VO-MO thin films may pose as promising candidates for use in futuristic silicon based solar cell and IR detector applications. Further, the reflectance data recorded in NIR region had, *in fact*, decreased with increase in film thickness, Fig. 4c.

On the other hand, the refractive index data of the VO-MO thin films on silicon substrate were calculated as a function of the RF power and are shown as inset of Fig. 4(c). The refractive index was found to be almost constant at about 2.9 to 3 (Fig. 4c, inset). These data (Fig. 4c, inset) compared very favourably with the recently reported refractive index of undoped V_2_O_5_ thin films on silicon^46^.

### Effect of annealing on VO-MO films

#### Microstructural study

**Figure 5:**
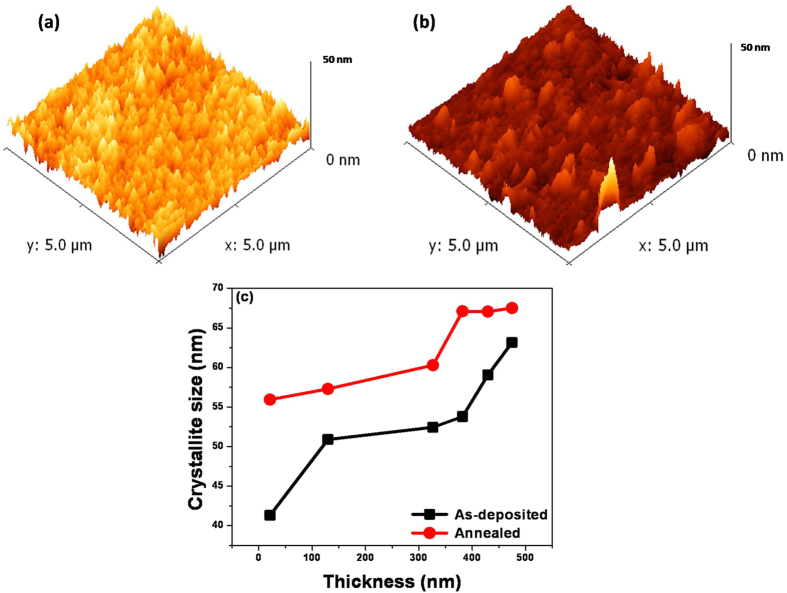
Typical AFM image of VO-MO film grown at 400 W/385 nm: (**a**) before (**b**) after annealing; and (**c**) variation of crystallite size as a function of film thickness both before and after annealing.

Figure 5(a,b) respectively show the AFM images of an as deposited 385 nm VO-MO thin film grown at an RF power of 400 W and that of the same film after annealing. The annealed film had the magnitude of R_a_ enhanced from 1.39 to 1.51 nm. As expected, the crystallite size increased from ~41 nm to ~63 nm as the VO-MO film thickness was increased from 21 nm to 475 nm (Fig. 5(c)). Thus, these data corroborated well with the XRD data (Fig. 2(c)) where the increase in relative intensity had occurred with increase in thickness; as discussed earlier. In addition, the annealed films exhibited further increase in crystallite size as shown in Fig. 5(c).

#### XPS investigation

**Figure 6:**
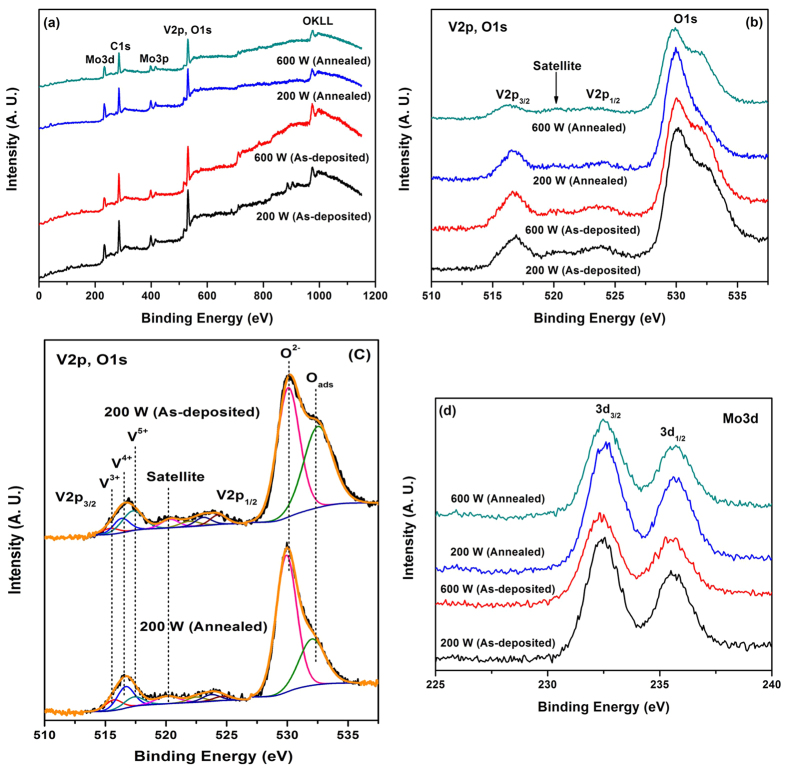
(**a**) Survey spectra (**b**) V2p and O1s spectra (**c**) curve-fitted V2p and O1s spectra; and (**d**) Mo3d core level spectra of VO−MO thin films with different conditions.

Figure 6(a) shows the XPS survey spectra of the VO−MO thin films grown at 200 and 600 W. Survey spectra of annealed thin films are also appended in the Fig. 6(a). The data from the survey spectra clearly showed the presence of V and O species in both as-deposited and annealed VO-MO thin films. The detailed XPS spectra of V2p core levels in as-deposited and annealed VO−MO thin films are presented in Fig. 6(b). Both V2p and O1s core level spectra are given as V2p and O1s core level regions are nearer to each other. Broad spectral envelopes of V2p core levels with long tail in the lower binding energy region indicated that V is present in different oxidation states and it can be curve-fitted into sets of spin-orbit doublets.

Accordingly, the observed V2p_3/2_ peaks at 515.4, 516.3 and 517.3 eV in the VO-MO thin films correspond to V^3+^ (V_2_O_3_), V^4+^ (VO_2_) and V^5+^ (V_2_O_5_) species that agrees well with the data reported in literature^9^,^15^,^47^. For 200 W RF power deposited VO-MO thin films in both as grown and as annealed conditions the typical curve-fitted V2p and O1s core level spectra are displayed in Fig. 6(c). Peak areas of V^3+^, V^4+^ and V^5+^ components were used to estimate their relative concentrations in the films^9^. Table 1 provides the data on the binding energies and relative surface concentrations of different V species as obtained from the V2p core levels of the as deposited and annealed VO−MO thin films grown with different RF powers.

The present VO−MO thin films showed presence of mixed oxides phases of vanadium^9^,^15^,^20^,^24^,^38^. The concentrations of V^4+^ and V^3+^ species in the films got enhanced after annealing at 100 °C. In particular, the O1s core level region was fitted with the two component peaks. The specific component peak at 530.0 eV was attributed to oxide species related to oxides, whereas the other specific component peak which had occurred at around ~532.0 eV was attributed to the presence of adsorbed oxygen in the coatings^15^. Mo3d core level spectra of these films are shown in Fig. 6(d). The observed Mo3d_5/2, 3/2_ core level peaks at 232.5 and 235.7 eV in all films were associated with Mo^6+^ species^15^,^48^.

#### Transmittance and reflectance properties

**Figure 7:**
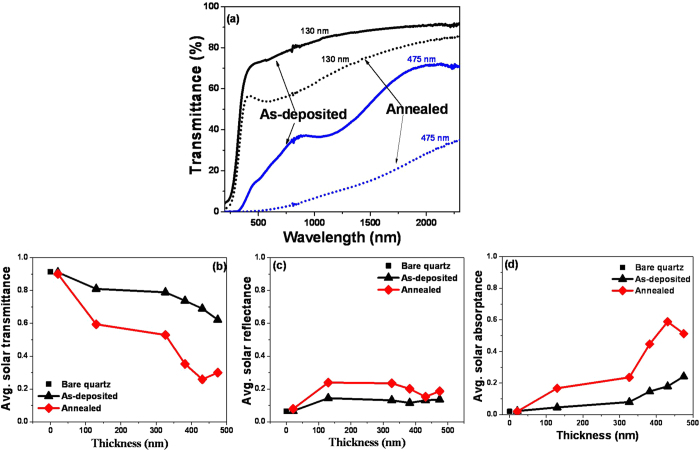
Effect of annealing on (**a**) transmittance spectra, average solar (**b**) transmittance, (**c**) reflectance and (**d**) absorptance data of VO-MO films on quartz.

As typical illustrative examples, the transmittance data of both relatively thin (e.g., 130 nm) and relatively thick (e.g., 475 nm) annealed VO-MO films on quartz substrates are shown in Fig. 7(a) as a function of wavelength. For the purpose of comparison only, the corresponding spectra of the as-deposited VO-MO films are also plotted in Fig. 7(a). As expected^42^,^49^, irrespective of thickness the transmittance values of the annealed VO-MO films were significantly lesser than those of the corresponding films in the as deposited conditions.

The data on variations of the average τ_s_, ρ_s_ and α_s_ of VO-MO thin films in both as deposited and as annealed conditions are shown in Fig. 7(b–d) as a function of film thickness. These films were deposited on quartz substrates, as mentioned earlier. Similarly, the experimental data presented in Fig. 7(b–d) were measured in the wavelength range of 200–2500 nm by using a reflectometer, as discussed earlier. The bare quartz showed the average α_s_ of ~2%, ρ_s_ of ~6.5% and τ_s_ of ~92%. With the increase in VO-MO film thickness, the average T_s_ had decreased while the average α_s_ and ρ_s_ had increased (Fig. 7(b–d)). Thus, the observations made in the present work were similar to the observation reported by others^9^,^29^.

The most interesting observation made in the present work, *however*, was that compared to those of the as deposited VO-MO films; the annealed VO-MO thin films had further reduced magnitudes of average τ_s_ as well as further enhanced magnitudes of the average ρ_s_ and α_s_. This observation was most likely linked to the fact that the annealed VO-MO thin films had surface roughness much higher than that of the as-deposited VO-MO thin films^42^,^43^.

#### Calculation of optical constants

**Figure 8:**
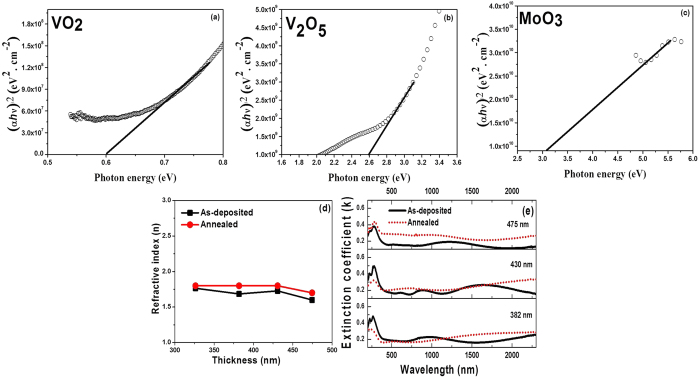
Typical ‘Tauc’ plots for calculating optical band gaps due to (**a**) VO_2_, (**b**) V_2_O_5_ and (**c**) MoO_3_ phases. Effect of annealing on (**d**) refractive index and (**e**) extinction coefficient of different VO-MO films as a function of thickness.

Typical ‘Tauc’ plots of (αhν)^2^ versus (hν) for the corresponding VO_2_, V_2_O_5_ and MoO_3_ phases are shown in Fig. 8(a–c), respectively. It is important to notice that due to mixed oxide phases of vanadium and molybdenum three different optical band gaps were observed in the present work, Fig. 8(a–c). This observation was also similar to those reported by others^22^,^50^.

The variation of the optical band gap of vanadium oxide and molybdenum oxide as a function of film thickness are summarized in Table 2. The present optical band gap data are well matched with those reported in literature for different oxidation state of vanadium oxide^50^,^51^,^52^ as well as molybdenum oxide^50^,^53^. Further, the data presented in Table 2 confirmed that the optical band gap decreased with the increase in VO-MO film thickness. These observations could be explained in terms of the well recognized quantum confinement or size effect^35^,^43^,^54^.

In addition, the annealed VO-MO thin films had the optical band gap data smaller than those of the as deposited VO-MO thin films (Table 2). This happened most likely because the annealed VO-MO thin films had enhanced surface roughness, Fig. 2(b), and crystallite size, Fig. 5(c). The decrease in the optical band gap has been linked^42^,^43^ with significant drop in transmittance which happens due to increase in grain size of the deposited vanadium oxide films. These observations (Table 2) were also well corroborated by the facts that the absorption edge had shifted towards higher wavelengths with increase in thickness, Fig. 4(a), as well as with post deposition annealing treatment, Fig. 7(a).

The variation of refractive index of VO-MO thin films on quartz substrate as a function of thickness is shown in Fig. 8(d). The data for films in both as deposited and as annealed conditions are included in Fig. 8(d). The refractive index of the as-deposited VO-MO thin films on quartz was almost constant at ~1.6–1.75 while after annealing it was marginally increased^49^ to e.g., ~1.7–1.8, Fig. 8(d). The minor differences in data could be due to the alteration of percentage of oxide species in the VO-MO thin films.

Further, the refractive index data (Fig. 8(d)) and reflectance spectra (Fig. 4(b)) were utilized to calculate thickness of the VO-MO films according to equation (1). Although not discussed earlier, these data were already included in Fig. 2(a). These data proved that the magnitudes of the theoretically calculated thickness were well matched with the thickness measured by the nanoprofilometry technique.

The variation of extinction coefficient (k) of the VO-MO films of different thickness is shown in Fig. 8(e) as a function of wavelength. The data for films in both as deposited and as annealed condition are included in Fig. 8(e). These films were deposited on quartz. The k values of the annealed films were always marginally higher than those of the as deposited films, Fig. 8(e). Thus, the present observations were similar to those reported by others^48^. These facts (Fig. 8(d)) also correlated well with the measured decrease in transmittance after annealing, Fig. 7(a).

#### IR emittance

**Figure 9:**
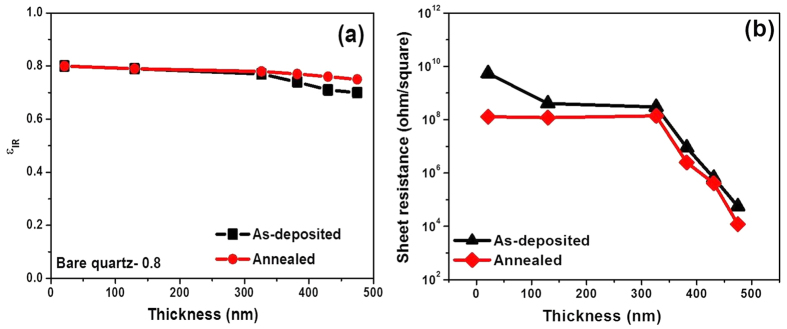
Effect of annealing on (**a**) IR emittance and (**b**) sheet resistance of different VO-MO films as a function of thickness.

The variation in ε_IR_ value of the VO-MO films is shown in Fig. 9(a) as a function of thickness. The data for films in both as deposited and as annealed conditions are included in Fig. 9(a). These films were deposited on quartz. The ε_IR_ data of the bare quartz substrate is also included in the same Fig. 9(a), for the purpose of comparison only.

The ε_IR_ value of bare quartz was measured as ~0.8, Fig. 9(a). At the lowest thickness of VO-MO i.e., 21 nm, the ε_IR_ data was not altered, Fig. 9(a). However, the ε_IR_ data was only marginally decreased to about 0.7 (Fig. 9(a)) with the increase in film thickness. Again, the annealed VO-MO thin films had ε_IR_ values marginally increased (Fig. 9(a)) over those of the as deposited VO-MO thin films.

#### Electrical property

The variation in R_s_ value of the VO-MO films is shown in Fig. 9(b) as a function of thickness. The data for films in both as deposited and as annealed condition are included in Fig. 9(b). These films were deposited on quartz. The R_s_ value of the as-deposited VO-MO thin films decreased from e.g., 5.5 × 10^9^ Ω/square to 5.6 × 10^4^ Ω/square with increase in thickness from ~21 nm to ~475 nm. The decrease in R_s_ as increase in film thickness is also reported for other oxide thin films such as indium tin oxide^39^.

The annealed VO-MO thin films showed R_s_ value lower than those of the as deposited VO-MO thin films, Fig. 9(b). The reduction in the R_s_ values was most likely linked to the increase in V^4+^ species after vacuum annealing as confirmed from the corresponding XPS investigations, Table 1. The reduction in oxidation state from V^5+^ to V^3+^ and V^4+^ caused an increase in the carrier density which led to further decrease in R_s_ of the annealed VO-MO thin films as compared to those of the as-deposited VO-MO thin films, Fig. 9(b).

#### Nanomechanical properties

The average E value of the as-deposited VO-MO film was measured as ~113.4 GPa. However, after annealing it was improved to e.g., ~135.1 GPa. The range of Young’s modulus data of vanadium oxide films reported in literature was really wide e.g., from about 5.6 GPa to 30 GPa^9^,^37^,^55^,^56^,^57^.

Similarly, the average H value of the as-deposited VO-MO thin films was measured as ~1.26 GPa. A much higher H value of ~2.14 GPa was measured for the annealed VO-MO thin films. The nanohardness of the present VO-MO thin films was significantly higher than the nanohardness value reported previously by us^9^,^37^ for VO thin films/coatings deposited on silicon substrates.

Therefore, the comparison of the E and H data from the present work with those reported earlier by us for VO thin films on silicon^9^,^37^ confirmed that significant enhancement in the nanomechanical properties of the VO thin films had definitely happened after incorporation of the suitable second phase e.g., molybdenum oxide. Further, the annealed VO-MO thin films had Young’s modulus increased by about 20% over that of the as deposited VO-MO thin films. Moreover, the annealed VO-MO thin films had nanohardness increased by about 70% as compared to that of the as deposited VO-MO thin films.

#### Combined FEM and nanoindentation studies

**Figure 10:**
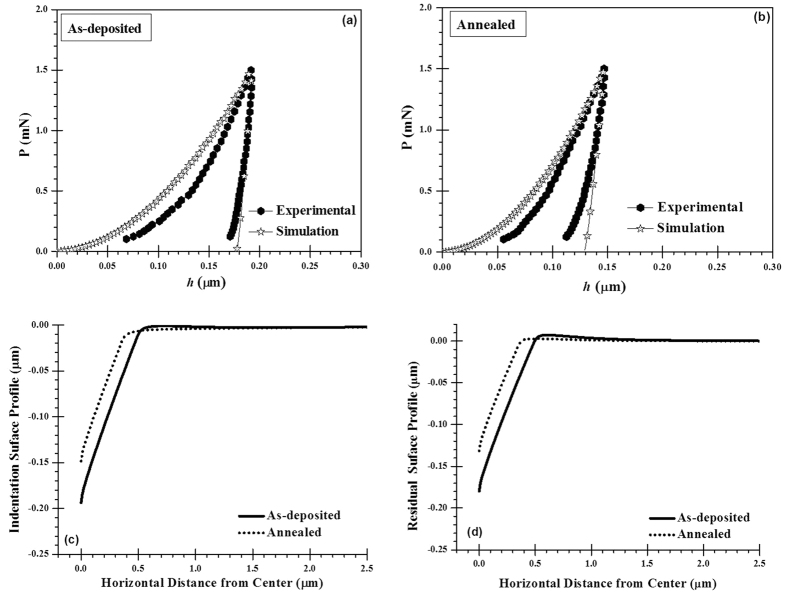
Comparisons of experimentally obtained and simulated P–h curves of (**a**) as-deposited and (**b**) annealed VO-MO films on quartz substrate. Indentation surface profiles during (**c**) maximum loading depth and (**d**) residual surface profiles after unloading for both as-deposited and annealed VO-MO films on quartz substrate.

Figure 10(a) shows the experimentally measured and FEM simulated *P*–*h* curves of the as-deposited VO-MO thin films on quartz substrates. Similar data for the corresponding annealed VO-MO thin films on quartz substrates are shown in Fig. 10(b). The experimentally measured and simulated *P*–*h* curves had a good match for the following specific combinations of plastic properties: as deposited VO-MO thin films (*σ*_*y*_ = 210 MPa and *n = *0.26) and annealed VO-MO thin films (*σ*_*y*_ = 412 MPa and *n = *0.31).

It can be seen from the *P–h* plots that at a given load of 1.5 mN, the experimentally measured depth of penetration in the annealed VO-MO thin film (Fig. 10(b)) was always smaller than that of the as deposited VO-MO thin film (Fig. 10(a)) on quartz substrate. This fact corroborated well with the improvement in nanomechanical properties of the annealed VO-MO thin films, as mentioned above.

For the as deposited and annealed VO-MO thin films, the simulated nanoindentation surface profiles during loading to the peak load of 1.5 mN are shown in Fig. 10(c) as a function of the horizontal distance from the center of the nanoindent. In an analogous manner, for the as deposited and annealed VO-MO thin films the simulated residual surface profiles during unloading from the peak load of 1.5 mN are shown in Fig. 10(d) as a function of the horizontal distance from the center of the nanoindent.

The profiles depicted in Fig. 10(c) reflected the conditions pertaining to the maximum penetration depth made by the nanoindenter during loading in the as-deposited and annealed VO-MO thin films. As expected, in both the films the zones of contact induced maximum deformations were predicted to occur just beneath the nanoindenter. Further, the depth of these contact induced deformation zones in both the films were predicted to continuously decrease with increase in distance from the center of the nanoindent until it would reach the surfaces of the corresponding films.

The residual surface profiles illustrated in Fig. 10(d) represented the conditions pertaining to the final penetration depth left by the nanoindenter during unloading from the peak loads of 1.5 mN those had been applied onto the as-deposited and annealed VO-MO thin films. For both the films the generic features of the simulated residual surface stress profiles (Fig. 10(d)) during unloading were similar to those of the simulated nanoindentation surface profiles during loading (Fig. 10(c)). The simulated nanoindentation surface profile shown in Fig. 10(c,d) also confirmed further that the possibilities of pile-up formations around the nanoindents in the as deposited and the annealed VO-MO thin films were non-existent.

**Figure 11:**
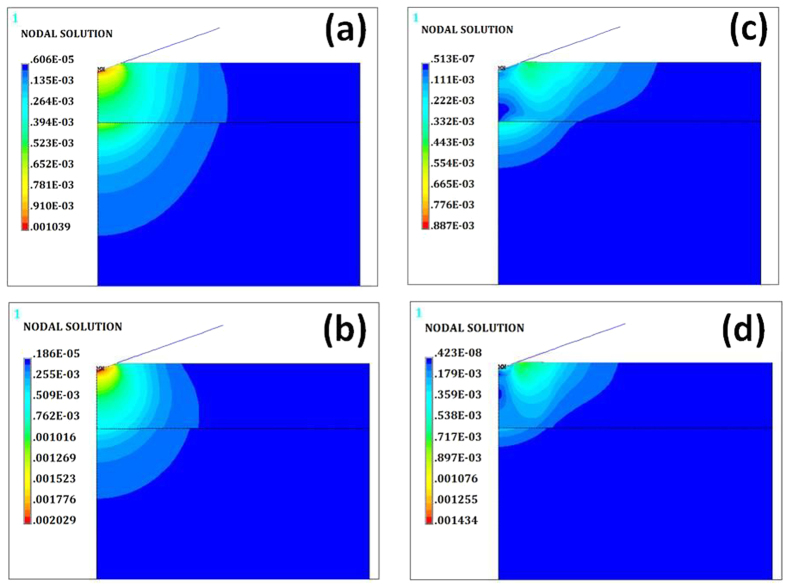
Development of von Mises stress distribution (in N.μm^−2^) in the as-deposited VO-MO film: (**a**) loading, (**c**) unloading and annealed VO-MO film: (**b**) loading, (**d**) unloading.

Figure 11(a) shows the distribution of von Mises stress for the as-deposited VO-MO thin films deposited on quartz substrate during loading. Similar data for the corresponding annealed VO-MO thin films during loading are shown in Fig. 11(b). Further, the distribution of von Mises stress for the as-deposited VO-MO thin films deposited on quartz substrate during unloading is shown in Fig. 11(c). Moreover, the similar data for the corresponding annealed VO-MO thin films during unloading are shown in Fig. 11(d).

Presumably owing to the high local stress concentrations linked with the sharpness of the nanoindenter tip (e.g., tip radius of about 150 nm), the FEM based simulations predicted that almost immediately after contact quite significant inelastic deformations would occur in the as-deposited (Fig. 11(a)) as well as the annealed (Fig. 11(b)) VO-MO thin films. The maximum von Mises stress for the as deposited and annealed VO-MO thin films on quartz substrate were estimated to be about 1.04 GPa (Fig. 11(a)) and 2.03 GPa (Fig. 11(b)). The relatively higher magnitude of stress developed in the annealed film reflected the greater intrinsic resistance of the film against nanoindentation induced contact deformation. This fact in turn corroborated well with the enhancement in nanomechanical properties in the annealed VO-MO thin films.

The von Mises stress acts as a hemispherical zone of plastic stress distribution inside both as deposited and annealed VO-MO thin films. After unloading, the hemispherical zone is released for both as-deposited and annealed VO-MO thin films (Fig. 11(c,d)). As a consequence, significant magnitudes of residual stress were predicted to exist just beneath the nanoindenter in both the films. Further, during unloading; the size of the residual stress distribution contour of the annealed VO-MO thin films (Fig. 11(d)) was predicted to be larger than that (Fig. 11(c)) of the as-deposited VO-MO thin films. The shapes of these residual stress distribution contours were, however, predicted to be irregular in shape for both as-deposited and annealed VO-MO films (Fig. 11(c,d)).

Due to the presence of such residual stresses the sizes of the zones of stress distribution contours during unloading (Fig. 11(c,d)) became smaller than those of the loading contours (Fig. 11(a,b)). This generic feature was true for both as-deposited (Fig. 11(a–c)) and annealed (Fig. 11(b–d)) VO-MO thin films deposited on quartz substrates.

#### The importance of smart behaviour of the present VO-MO films

As discussed earlier, pure or undoped VO_2_ will show reversible or smart transition temperature at 68 °C^32^. However, the reduction of transition temperature (i.e., close to room temperature) is always beneficiary from the application point of view. That is in fact why several attempts are reported towards the reduction of transition temperature of VO_2_ with doping/adding a transition metal or a second phase^14^,^15^,^17^,^18^,^19^,^20^,^24^,^25^,^31^.

In the present study, introduction of molybdenum oxide gives much lower transition temperature i.e., around 45–50 °C (Fig. 3) as compared to the transition temperature (i.e., 68 °C)^32^ of pure (i.e., undoped) VO_2_. Thus, the addition of molybdenum oxide is proven beneficiary from the smart behaviour point of view. In fact, the reduction of transition temperature will be much desired for smart radiative device^58^ (i.e., tunable emittance surface) in spacecraft thermal control application as various elements of electronic subsystem in general can operate up to aforesaid temperature region i.e., 45–50 °C. However, various elements of electronic subsystem of spacecraft do not work beyond 50 °C. Thus, for this specific application, pure VO_2_ will not serve the purpose as it shows the transition temperature much beyond 50 °C (i.e., at 68 °C).

#### Superior nanomechanical properties of present nanocolumnar and crystalline VO-MO film

The improvements in nanomechanical properties are also obtained after inclusion of second phase as molybdenum oxide. The average Young’s modulus value of the as-deposited VO-MO film is measured as ~113.4 GPa. This value of Young’s modulus is much higher than the band (e.g., 5.6–30 GPa) of Young’s modulus data generally reported for undoped vanadium oxide films^9^,^37^,^55^,^56^,^57^. In a few instances, however, depending on the specific plane of growth such as along (011)^59^, (200)^60^ and b-axis^61^ relatively higher modulus values e.g., 120 GPa^59^, 129 GPa^60^ and 220 GPa^61^ are also reported for undoped vanadium oxides.

The nanohardness of VO-MO thin film is measured in the present work to be as high as 1.26 GPa. This nanohardness is 6.3 times higher than the nanohardness of 0.2 GPa reported earlier by us^9^,^37^, for the undoped sputtered vanadium oxide thin films deposited on silicon substrates. Further, the nanohardness values of pure molybdenum oxide films are reported to be up to as high as e.g., 1.34 GPa^62^ and 2.4 GPa^63^. These data were of the same order of magnitude as the nanohardness of the present VO-MO thin films. In addition, the Young’s modulus of bulk molybdenum oxide is much higher i.e., 540 GPa as compared to that of the vanadium oxide. The high magnitude of the Young’s modulus of the bulk molybdenum oxide could have also contributed to the relatively higher Young’s modulus of 113.4 GPa measured for the present VO-MO thin film. Thus, the data from the present work on VO-MO thin film achieved a significant, noteworthy improvement in the nanomechanical properties as well as in the intrinsic physical resistance against nano scale contact induced deformation. It is almost needless to mention that the superior mechanical integrity is always preferred in general when such VO-MO thin film based device will be in-service.

It is already well known that the crystalline ceramic always possess better mechanical integrity as compared to those of the materials which belong to the amorphous state. It needs to be recalled that the present pulsed RF sputtering technique offers a nanocolumnar structure (Fig. 1c) of the VO-MO film. In general, nanostructures always show superior mechanical properties as compared to those exhibited by the relatively coarse grain microstructures. Further, as shown earlier in the data presented in Fig. 2(c), the present VO-MO thin films covering the entire thickness range of about 21 nm to 475 nm are proved to be highly crystalline. Therefore, by virtue of possessing this highly crystalline nanostructure the present nanocolumnar and crystalline VO-MO thin films possess superior mechanical properties.

#### Trade-off on optical and electrical properties of the present VO-MO film

It is very interesting to note that without significant alteration of phase and oxidation state (Fig. 2(c) and Table 1), the optical property i.e., solar transmittance can be tuned from 91% to 62% (Fig. 7b) just by altering the thickness of the as deposited VO-MO thin films from 21nm to 475 nm. Further, the RF sputtered undoped vanadium oxide film shows transmittance property^9^ comparable to that (Fig. 7(b)) obtained in the present work.

The solar transmittance value of sol-gel based undoped VO_2_ is reported^64^ to be about 60.5%. The transmittance is marginally dropped up to 56% after incorporating maximum percentage (e.g., 3%) of W^64^. On the contrary, both decrement and increment of optical transmittance are reported after incorporation of Mo and Mn in VO_2_^15^. Thus, in the present study, the addition of molybdenum oxide in vanadium oxide thin films achieves the main purpose of a significant reduction in the transition temperature along with the huge improvement in nanomechanical properties, as described earlier.

On the other hand, the sheet resistance of VO-MO thin films decrease with increase in thickness of the film as shown earlier in Fig. 9(b). It is believed that the increase in thickness improves crystallinity of the film and hence increases the density of charge carriers^39^,^65^. Thus, the resistance of the film is decreased with the increase in film thickness.

Further, it is true for an oxide film/coating that the optical transmittance and electrical conductivity always behave in opposite manners. Thus, a film that is optically highly transparent will show comparatively lower electrical conductivity as compared to that of the thicker film where transmittance will be degraded. In the present case, at low thickness regime (i.e., 21 nm to 326 nm), sheet resistance value (Fig. 9(b)) is in the range of 10^9^ to 10^8^ Ω/square with a correspondingly high optical transparency (i.e., 91 to 79%).

Finally, at the highest thickness of the VO-MO films i.e., 474 nm, the sheet resistance drops to the lowest magnitude of e.g., 10^4^ Ω/square (Fig. 9(b)). Such a huge drop obviously signifies a concomitant improvement in conductivity along with the achievement of a moderate average solar transmittance of ~62%. Thus, the VO-MO films developed in the present work offer a scope that depending on the demand imposed by the prospective application scenario it could be possible to tune the optical transmittance over a wide range e.g., 62% to 91% along with the correspondingly wide range of sheet resistance e.g., 10^4^ Ω/square to 10^9^ Ω/square. In other words, the data of the present work provide a scope to tune the optical transparency and electrical conductivity of the VO-MO thin films as per demand of the chosen application.

#### Novel antireflectance behaviour of VO-MO films for futuristic silicon based solar cell

In the present work, VO-MO thin films deposited on both quartz and silicon substrates show low reflectance properties (Fig. 7(b,c)). But, one of the most interesting observations of the present work is that the VO-MO thin films on silicon substrates exhibit a reduction in reflectance in particularly the visible region (Fig. 7(c)). The extent of reduction was much more than that of the silicon substrate in the same visible region (Fig. 7(c)). These data (Fig. 7(c)) strongly suggests that the present VO-MO thin films deposited on silicon substrates exhibit the antireflective characteristics. This novel antireflection property of the VO-MO thin films deposited on silicon substrates can be utilized towards the application of silicon based energy harvesting devices.

These VO-MO thin films deposited on silicon substrates are proposed to be anti-reflective because they exhibit (Fig. 7(c)) reflectance much lower than that of the silicon substrate. As silicon is a fundamental device component of all solar cell applications involving the exploitation of the antireflection property, the present work is done with a judicious choice to use silicon as the substrate such that the antireflection property of the present VO-MO thin films can be amply demonstrated.

In silicon based solar cell, monolayer, bi-layer or multilayer coatings of various ceramics materials such as TiO_2_, SiO_2_, Al_2_O_3_, Si_3_N_4_ are often used as antireflective layer^66^. Further, the effective antireflective behaviour can be achieved by applying surface texturing^67^ and by combination of antireflection layers with surface texturing as well^68^. However, these technologies are not only expensive but also too complex in nature which results in very limited utilization of the same for the practical application.

Thus, the uniqueness of the present work lies in the fact that it demonstrates for the very first time that only a single thin film of VO-MO on the silicon substrate can be utilized as an antireflective layer. In addition to its antireflective property the VO-MO thin films also show the smart phase transition to occur reproducibly at a much lower transition temperature of around 45 to 50 °C. This is further beneficiary due to its inherent variable IR emittance behaviour. It is therefore plausible to argue that due to its inherent variable IR emittance, the VO-MO thin films may assist to release the excess heat energy generated by the silicon based solar cell device *in-operation*. The increase in cell temperature can further cause lower efficiency and life^69^.

#### IR transparency behaviour of the present VO-MO films

As discussed earlier, irrespective of its thickness the as-deposited VO-MO films show insignificant change (~0.8 to 0.7) in IR emittance property with respect to that (e.g., ~0.8) of the bare quartz substrate (Fig. 9a). This information signifies that the present VO-MO films are IR transparent like the undoped vanadium oxide films^9^. Thus, even after adding a second phase such as molybdenum oxide, the characteristic behaviour of the undoped vanadium oxide is not changed^9^. Thus, this IR transparency property of the present VO-MO films can pose them as a potential candidate in applications such as IR detector and IR imaging.

#### Impact of low temperature vacuum annealing on structural, functional and mechanical properties

It is very interesting to notice that the concentrations of V^4+^ (i.e., VO_2_) and V^3+^ (i.e., V_2_O_3_) species in the VO-MO thin films got enhanced after vacuum annealing at 100 °C as mentioned earlier (Table 1 and Fig. 6). Actually, the dissociation of V_2_O_5_ gives rise to lower valence oxides of vanadium^52^. Consequently, after vacuum annealing, decrease in R_s_ value is measured (Fig. 3(b)). It is obvious that the decrease in R_s_ value is linked with increase in V^4+^ species after vacuum annealing as found from the XPS investigation (Table 1 and Fig. 6).

The reduction in oxidation state from V^5+^ to V^3+^ and V^4+^ causes an increase in carrier density. This process leads to further reduction in the R_s_ values of the annealed VO-MO thin films as compared to those of the as deposited VO-MO films (Fig. 9b). For instance, the thickest i.e., 475 nm VO-MO film exhibits the R_s_ values of 56 kΩ/square and 15 kΩ/square respectively in as deposited and vacuum annealed conditions. In this particular context, it is important to mention that this range of R_s_ value of vanadium oxide is reported to be suitable for bolometer applications^70^.

Further, the solar transmittance of the vacuum annealed VO-MO thin films is smaller than that of the as deposited VO-MO thin film (Fig. 7(b)). As discussed earlier this occurs primarily due to the increase in surface roughness (Fig. 5a,b) and crystallite size (Fig. 5c) of the film after vacuum annealing. The vacuum annealing further results in a decrease in optical band gap (Table 2).

However, the IR emittance value (e.g., ~0.8) of the annealed VO-MO film is almost similar to those of the as deposited film (0.8–0.7) as well as the quartz substrate (~0.8), Fig. 9(a). This data trend suggests that the present VO-MO films retain their IR transparency. This retention of IR transparency property even after annealing is very important for two major applications. The first is that for any given IR detector or imaging device. The second is that of a tunable emittance coating for spacecraft thermal control. It is important to mention in this particular context that because of the inherent smart phase transition capability at low temperature the vanadium oxide based materials can offer the IR transparency and at temperatures beyond phase transition temperature the vanadium oxide based materials can offer the IR opaque characteristics^70^,^71^, thereby justifying possibility of smart radiative surface.

Finally, superior mechanical integrity of a thin film is always an important criterion when it will be in-service condition. Here, it is noticed that the both nanohardness (1.26 GPa vs. 2.14 GPa) and Young’s modulus (113.4 GPa vs. 135.1 GPa) of as-deposited VO-MO film are significantly improved after the annealing. Further, combined FEM with nanoindentation approach predicts the values of yield stress of the as-deposited VO-MO film as 210 MPa which is also improved to 412 MPa. The increase in crystallinity due to annealing^72^,^73^ is believed to be the reason behind the improvement of mechanical properties of the present VO-MO films.

## Conclusions

Nanocolumnar, crystalline and uniform vanadium oxide-molybdenum oxide thin (21 to 475 nm) films were grown by pulsed RF magnetron sputtering technique. The average surface roughness and crystallite size of the VO-MO thin films increased with increase in thickness. Both roughness and crystallite size of VO-MO thin films was further increased after annealing. The reduction in transmittance occurred with increase in film thickness. Further, a noteworthy drop in the transmittance was observed after annealing. The reflectance data of VO-MO coated silicon substrates were measured to be always lower than that of the bare silicon substrate. These data signified the characteristic presence of antireflection behaviour. Both DSC and temperature dependent sheet resistance data showed smart i.e., reversible phase transition at the transition temperature of around 45–50 °C. The dual optical band gaps corresponded to presence of VO_2_ and V_2_O_5_ in the present work. Due to the presence of the MoO_3_ phase a relatively higher optical band gap was measured at the corresponding higher photon energy. The optical band gaps decreased with increase in film thickness. Further, the optical band gaps decreased after annealing. It happened possibly due to the increase in surface roughness and crystallite size in the annealed VO-MO thin films. The refractive index of the as-deposited VO-MO thin film on quartz was almost constant at about 1.6–1.75 and it marginally increased to 1.7–1.8 after annealing. However, the refractive index of VO-MO film on silicon is calculated as around 3. The present VO-MO thin films had nanomechanical properties much superior to those reported in literature for VO thin films. The average nanohardness and Young’s modulus were measured respectively as ~1.26 GPa and ~113.4 GPa for the as deposited VO-MO films. Further, the annealed VO-MO thin films had Young’s modulus and nanohardness increased in correspondence by about 20% and 70% over those of the as deposited VO-MO thin films.

## Additional Information

**How to cite this article**: Dey, A. *et al.* Nanocolumnar Crystalline Vanadium Oxide-Molybdenum Oxide Antireflective Smart Thin Films with Superior Nanomechanical Properties. *Sci. Rep.* **6**, 36811; doi: 10.1038/srep36811 (2016).

**Publisher’s note**: Springer Nature remains neutral with regard to jurisdictional claims in published maps and institutional affiliations.

## Figures and Tables

**Table 1:** Binding energies and relative peak areas of V species in VO−MO thin films evaluated from XPS studies.

Details of VO-MO films grown at different conditions	V species	Binding energy of V2p_3/2_ (eV)	Relative peak area (%)
130 nm/200 W (As-deposited)	V^3+^	515.4	13
	V^4+^	516.3	31
	V^5+^	517.3	56
130 nm/200 W (Annealed)	V^3+^	515.6	24
	V^4+^	516.3	50
	V^5+^	517.4	26
475 nm/600 W (As-deposited)	V^3+^	515.5	14
	V^4+^	516.5	38
	V^5+^	517.1	48
475 nm/600 W (Annealed)	V^3+^	515.3	29
	V^4+^	516.4	44
	V^5+^	517.1	27

**Table 2:** Optical band gaps of as-deposited and annealed VO-MO films due to VO_2_, V_2_O_5_, and MoO_3_ phases.

Thickness (nm)	VO_2_ (eV)		V_2_O_5_ (eV)		MoO_3_ (eV)	
	As-deposited	Annealed	As- deposited	Annealed	As- deposited	Annealed
21	0.7	0.69	2.8	2.7	3.46	3.3
130	0.68	0.68	2.68	2.56	3.45	3.4
326	0.65	0.64	2.61	2.52	3.3	3.36
382	0.62	0.6	2.56	2.5	3.15	3.15
430	0.58	0.6	2.5	2.5	3.18	3.1
475	0.6	0.56	2.5	2.46	3.07	3
